# Electrically Tunable Metalens Based on PEDOT:PSS

**DOI:** 10.3390/mi16121341

**Published:** 2025-11-27

**Authors:** Miao Zhang, Dizhi Sun, Shiqi Zhang, Liangui Deng, Jiaxin Li, Jianguo Guan

**Affiliations:** 1 State Key Laboratory of Advanced Technology for Materials Synthesis and Processing, Wuhan University of Technology, Wuhan 430070, China; 2 School of Information Engineering, Wuhan University of Technology, Wuhan 430070, China; 3 China Electric Power Research Institute, Beijing 100192, China

**Keywords:** metalens, PEDOT:PSS, reconfigurable manipulation

## Abstract

Tunable metalenses are planar optical elements that hold immense potential in the field of integrated optics, enabling reconfigurable focusing without the bulkiness associated with traditional lenses. This study proposes an electrically tunable metalens which integrates poly(3,4-ethylenedioxythiophene)–polystyrenesulfonate (PEDOT:PSS) with a metasurface. The focal length is electrically controlled through electrochemical modulation of the PEDOT:PSS film thickness and deintercalation in an electrolyte. The Fresnel zone plate (FZP) design is employed to simplify the phase profile and reduce optimization complexity. More importantly, the modulated PSO algorithm is implemented to inverse-design the units and suppress inter-unit phase crosstalk. Simulation results demonstrate that the metalens achieves diffraction-limited focusing, with a zoom ratio reaching 10:1. This work provides a feasible strategy for developing high-performance dynamically tunable metalens, with promising applications in miniaturized imaging, microscopy, and integrated photonic systems.

## 1. Introduction

Tunable lenses are a core technology with wide applications in imaging systems [1,2], optical communications [3], and laser processing [4,5]. Conventional zoom systems rely on mechanical adjustments of the lens spacing to vary focal length, which often results in bulky and complex setups. Metasurfaces [6,7,8,9,10,11,12,13,14,15], composed of subwavelength nano-units, offer a promising alternative by enabling precise control over the amplitude, phase, and polarization of light within an ultra-thin platform. Among their most prominent applications, metalenses stand out due to their high integration density and multifunctional capabilities compared to conventional refractive optics. These devices have already demonstrated potential across various fields, including beam focusing [16], edge detection [17], and hyperspectral imaging [18]. However, the focal length of a typical metalens is fixed upon fabrication, which has motivated extensive research toward realizing tunable metalenses.

To achieve dynamic wavefront control in metalenses, various material-based tuning mechanisms have been extensively explored. These include vanadium dioxide (VO_2_), which utilizes a thermal phase transition to enable functions ranging from simple focus switching to complex continuous zoom and multi-focus switching [19,20]. Liquid crystals integrate with dielectric metasurfaces for focal length switching via electrical polarization control [21,22,23,24,25,26] and chalcogenide phase-change materials (GSST) that switch between amorphous and crystalline states to deliver bifocal functionality [27]. Recently, Poly(3,4-ethylenedioxythiophene)–polystyrenesulfonate (PEDOT:PSS) has emerged as a promising material for dynamic metasurfaces, distinguished by its remarkable environmental stability, tunable optoelectronic properties, excellent electrical conductivity, and high modulation depth [28,29,30]. The integration of conducting polymers with metasurfaces for dynamic control generally follows two distinct approaches [31]. The first strategy employs PEDOT:PSS as an intrinsically resonant material in the form of directly patterned nano-units. This approach leverages the metal–insulator transition characteristic of PEDOT:PSS in the near-infrared region, enabling reversible switching of plasmonic resonances [32,33,34,35,36], thus realizing electrically switchable beam steering [32] and gradually tunable nano-optical antennas [33]. However, such all-polymer metasurfaces have so far been largely confined to the infrared regime, limited by the inherent carrier density and mobility of PEDOT:PSS [31]. Extending their operation to visible wavelengths requires achieving carrier densities exceeding 1 × 10^22^ cm^−3^ while maintaining high mobility [36], which remains an unresolved material challenge. The second scheme integrates conducting polymers with resonant antennas to achieve enhanced tunability while overcoming the limitations of all-organic structures [29,31,37,38,39,40]. This strategy has yielded demonstrations of focal intensity modulation exceeding 90% contrast via electrochromic polymer–gold nanorod integration [39], and most recently, flexible metasurfaces exploiting PEDOT:PSS swelling for high-efficiency beam control at CMOS-compatible voltages [29]. However, current approaches often rely on a direct mapping from the structure to the electromagnetic response within a pre-defined library. This method typically implements simple phase patterns and has a limited functional scope. Consequently, generating irregular and reconfigurable phase slopes remains challenging, hindering the realization of more complex devices such as metalenses.

**Figure 1:**
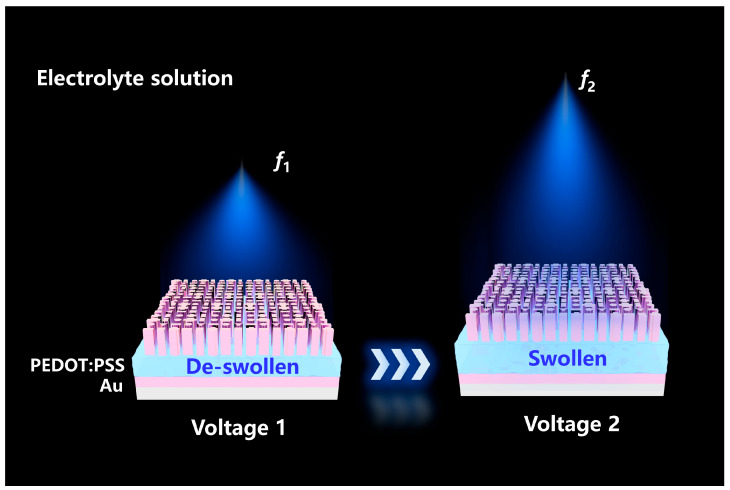
Electrically tunable metalens with PEDOT:PSS showing how applied voltages (Voltage 1 or 2) control the swelling state of the PEDOT:PSS layer within the electrolyte solution, leading to a shift in the metalens focal point from *f*_1_ to *f*_2_.

In this work, we utilize an inverse design strategy using modulated particle swarm optimization (PSO) to map the PEDOT:PSS-enabled reconfigurable phase gradient onto the required metalens phase profile. As illustrated in Figure 1, the device utilizes the high electrical conductivity of PEDOT:PSS to drive the intercalation of electrolyte ions through electrochemical means. The resultant swelling and de-swelling of the polymer film modulate the optical path length of the Fabry–Pérot (FP) cavity, facilitating reconfigurable phase modulation. By elaborately tuning the geometry and size of the nano-units, we build an electromagnetic response library of Au nano-units with distinguished phase gradients. More importantly, a PSO algorithm is employed to accurately map target phase distributions to corresponding structural parameters in the library, reducing the average phase error per unit cell to 0.81, thus enhancing the focusing performance of the tunable metalens. Simulation results validate that the designed metalens operates as a diffraction-limited tunable metalens, exhibiting dual-focal capabilities and a 10:1 zoom ratio.

This research aims to provide a new technical pathway for the application of dynamic metalenses in the visible band, with promising implications in fields such as miniaturized imaging systems and tunable optical communication devices.

## 2. Results

### 2.1. Operating Principle of the PEDOT:PSS-Based Metalens

**Figure 2:**
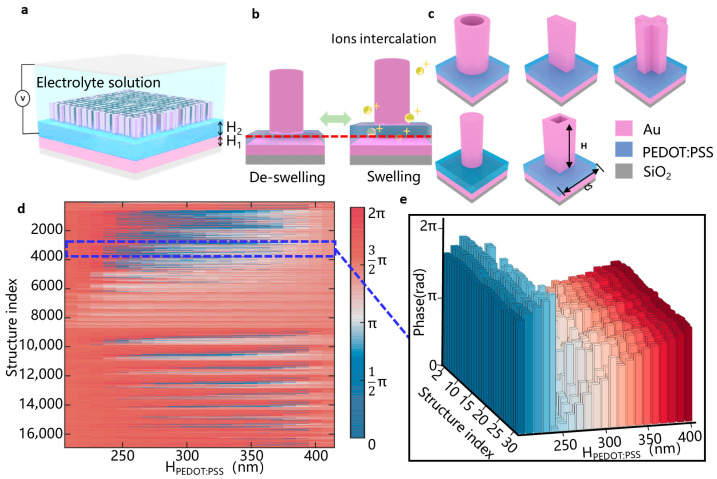
Dynamic mechanism of voltage-tunable metalens. (**a**) The electrically tunable metalens structure; (**b**) voltage changes induce intercalation of ions from the electrolyte solution into the PEDOT:PSS layer, thereby causing the swelling and de-swelling of the layer; (**c**) geometry of the constituent nano-unit; (**d**) corresponding phase response library mapping the unit cell’s phase shift to its PEDOT:PSS thickness H_PEDOT:PSS_; (**e**) detailed view of the phase distribution in (**d**), demonstrating a full 0–2π phase coverage, with the color change representing H_PEDOT:PSS_.

Figure 2 shows the schematic of the dynamic mechanism of voltage-tunable metasurfaces. As shown in Figure 2a, the device consists of a multilayer structure built on a SiO_2_ substrate, incorporating a 100 nm thick Au bottom mirror that defines one end of an FP cavity. A 200 nm thick PEDOT:PSS layer serves as the electrochemically dynamic medium. This functional polymer layer is capped with a Au nano-unit with a 360 nm period and 70 nm height. The entire structure is immersed in an electrolyte, completed by an ITO top electrode to form a fully integrated electrochemical cell. A uniform dielectric medium is employed in the simulation to represent the tetrabutylammonium hexafluorophosphate (TBAPF_6_) in acetonitrile (ACN) electrolyte solution, with a typical concentration of 1 mol/L [28]. This well-established system demonstrates excellent electrochemical stability and compatibility with PEDOT:PSS, which exhibits a switching response on the order of seconds and reliable cycling stability, thereby ensuring reproducible device operation [28,29].

The dynamic tuning mechanism, depicted in Figure 2b, relies on the electrochemical response of PEDOT:PSS to applied voltages. The PEDOT:PSS polymer layer contains fixed negative PSS^−^ charges. When a negative voltage is applied, large positive ions from the electrolyte solution migrate into the PEDOT:PSS layer to compensate for the negative charges, causing the polymer layer to swell. Conversely, when a positive voltage is applied, the positive ions are expelled from the polymer layer, resulting in film shrinking. This electrochemical responsiveness enables the PEDOT:PSS layer to achieve a twofold change in its thickness as the applied voltage varies [29]. This thickness variation directly alters the cavity length of the FP cavity, leading to modulations in the transmission phase, which, through its constructive and destructive interference with the phase of the nano-units’ plasmonic resonance, enables precise control over the final complex amplitude. This behavior aligns well with the theoretical phase accumulation in an FP cavity, which can be expressed as:

(1) $${∆\varphi }_{PEDOT:PSS}=\frac{2\pi }{\lambda }\times n\times ∆h_{PEDOT:PSS}$$

where ${∆\varphi }_{PEDOT:PSS}$ is the phase accumulated by the light transportation in PEDOT:PSS, $∆h_{PEDOT:PSS}$ is the thickness variation, *n* is the refractive index of PEDOT:PSS, and *λ* is the operating wavelength. The combination of this phase–thickness relationship and the geometric diversity of the nano-units provides a wide range of phase options, enabling accurate approximation of the target wavefront.

We constructed a comprehensive electromagnetic response library by utilizing five distinct Au nano-unit geometries (including cuboids, cylinders, toroids, rectangular rings, and crosses), as presented in Figure 2c. As shown in Figure 2d, the response library contains 355,782 datasets encompassing 16,942 nano-unit configurations across 21 distinct PEDOT:PSS thicknesses ranging from 200 to 400 nm, which can be dynamically modulated with voltage from −1.5 v to +1.5 v. This library enables continuous phase coverage ranging from 0 to 2π, which is realized through systematic variations in the geometry and dimensions of the Au nano-units, as well as adjustments to the thickness of the PEDOT:PSS layer. The partial structures index from the full library is illustrated in Figure 2e (indices 3240–3270); a continuous phase shift covering the full 0–2π range is achieved by varying the PEDOT:PSS thickness (H_PEDOT:PSS_), as indicated by the color transition from blue to red. Furthermore, a phase gradient can also be introduced by varying the lateral dimensions of the nano-units, providing an additional degree of freedom for wavefront control.

### 2.2. Design Process of the PEDOT:PSS-Based Metalens

For a metalens, the phase distribution corresponding to a specific focal length *f* can be expressed as follows:

(2) $$\varphi_{theoretical}=-\frac{2\pi }{\lambda }\left(\sqrt{x^{2}+y^{2}+f^{2}}-f\right)+C$$

In this formula, *λ* denotes the target wavelength of the incident light, *f* represents the focal length of the metalens, and *x* and *y* coordinates indicate the spatial position of each unit on the metasurface. The term C is a constant phase offset. While its inclusion does not alter the phase gradient, it introduces a global phase shift that facilitates the metalens design by enabling a closer match between the theoretical phase profile and the discrete phases available in the electromagnetic response library of the nano-units. In this work, a Fresnel zone plate (FZP) design strategy is employed to optimize the focusing efficiency of the metalens, which partitions the metalens into concentric annular regions, comprising main zones (m), each further subdivided into sub-zones (n). This zonal design approach offers a significant reduction in the number of phase variables, thereby decreasing the types of nano-units required for fabrication and lowering the optimization complexity.

**Figure 3:**
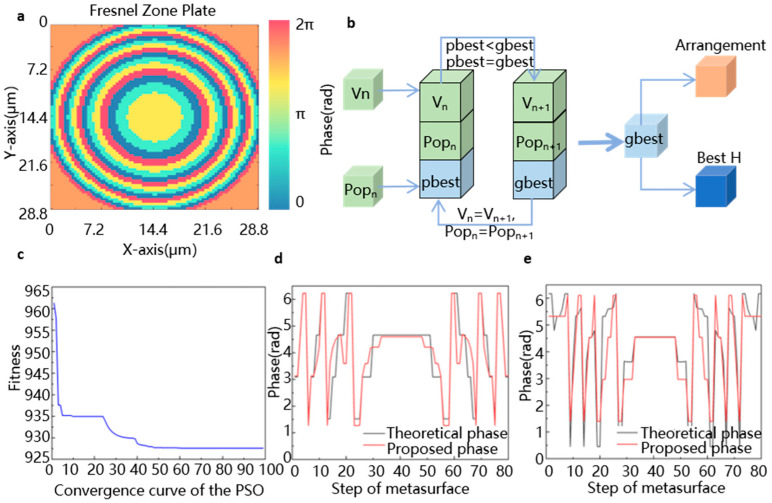
The design process of the PEDOT:PSS-based metalens. (**a**) Metalens Fresnel zone plate design; (**b**) metasurface design and optimization process; (**c**) relationship between number of iterations and fitness; (**d**) comparison between theoretical phase and proposed phase under focal length 1; (**e**) comparison between theoretical phase and proposed phase under focal length 2.

Figure 3 schematically illustrates the design process of our dynamic metalens. Based on the target application scenario, we confirmed the working wavelength *λ* = 589 nm, target focal lengths (*f*_1_ = 30 μm, *f*_2_ = 50 μm), lens radius set to 14.4 μm, and the number of main zones (m = 4) and sub-zones (n = 4). The zonal configuration is depicted in Figure 3a. The modified PSO algorithm is configured to minimize the phase error by optimizing the constant phase offset C in Equation (1). This adjustment shifts the theoretical phase profile to better align with the real electromagnetic responses in the nano-unit library, thereby determining the optimal arrangement of each nano-unit and the corresponding PEDOT:PSS thickness. The detailed PSO workflow is illustrated in Figure 3b. Herein the loss function is defined as

(3) $$L_{i}=\sum_{x=1}^{p}\sum_{y=1}^{p}\left|\varphi_{T,i}\left(x,y\right)-\varphi_{P,i}(x,y)\right|$$

(4) $$L=L_{1}+L_{2}+0.1\left|L_{1}-L_{2}\right|$$

where $\varphi_{T}(x,y)$ is the theoretical phase at position (x,y), $\varphi_{p}(x,y)$ is the phase retrieved from the response library, and p denotes the step of metalens. This loss function simultaneously evaluates both the phase difference between the theoretical and proposed phase profiles selected from the response library and the absolute difference in total phase deviation between the two target focal lengths. This dual consideration ensures a balanced imaging performance for the tunable metalens across its operating range.

During iterations, each particle tracks its own historical best position (pbest), corresponding to the constant phase *C*, while the entire swarm collectively identifies the global best position (gbest) found by any particle, as shown in Figure 3b. Through this iterative process, the algorithm converges toward the parameter combination that minimizes the total loss. The final output provides the structural layout and the optimal PEDOT:PSS thickness (*H*), yielding an electrically controlled metalens that integrates high phase accuracy with balanced multi-focal performance. As shown in Figure 3c, the fitness value converges to a stable minimum as iterations proceed, indicating that the nano-unit array has reached its optimal configuration. As shown in Figure 3d,e, the designed phase profiles exhibit excellent agreement with their theoretical counterparts, demonstrating high phase modulation accuracy for both focal lengths of *f*_1_ and *f*_2_.

Through the above design process, we overcome the limitation of relying on a pre-defined structural library. This approach enables the generation of complex and reconfigurable phase profiles that are essential for high-performance tunable metalenses.

### 2.3. Simulation of the Electrically Tunable Metalens

**Figure 4:**
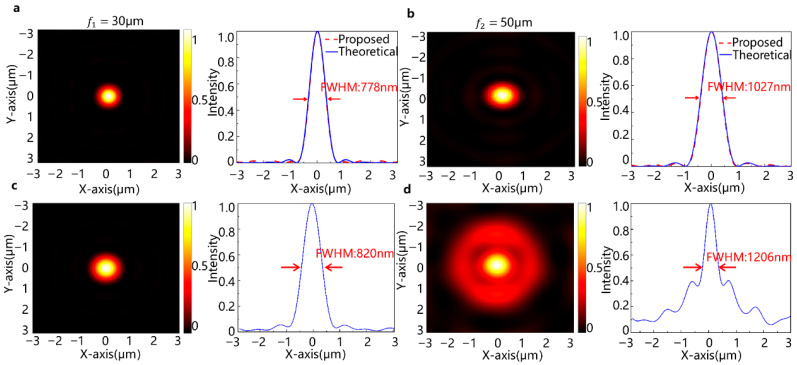
Simulation results and analysis at focal length 30 μm and 50 μm. (**a**) The right side shows the focused spot images and (left) the x-sections of the z = 30 μm focal line image at HPEDOT = 230 nm; (**b**) same as (**a**) but for z = 50 μm and HPEDOT = 350 nm; (**c**,**d**) the focal spot images and cross-sectional profiles under the same conditions as (**a**) and (**b**), respectively.

To clearly validate the electrically tunable focusing performance of the proposed design, simulations are conducted using both Kirchhoff’s diffraction integral and the Finite-Difference Time-Domain (FDTD) method. Figure 4a,b show the optical field distributions calculated based on Kirchhoff’s diffraction theory, which fully accounts for the contribution of all points on the incident surface to the detection points, reproducing the physical process of light propagation. As shown in Figure 4a, under Voltage 1 with a PEDOT:PSS thickness of 230 nm, the metalens focuses at z = 30 μm, yielding a full width at half maximum (FWHM) of 778 nm. When the voltage is switched, increasing the PEDOT:PSS thickness to 350 nm, the focal point shifts to z = 50 μm with an FWHM of 1027 nm, demonstrated in Figure 4b. For comparison, Figure 4c,d present optical field distributions calculated through a two-step process: FDTD simulations provided the near-field response over a 29.8 × 29.8 μm detection plane, followed by scalar diffraction propagation calculations that simulated field propagation to the far-field region. This approach ensures accurate near-field characterization while enabling efficient analysis of large propagation distances. The FWHM values from FDTD are 820 nm and 1206 nm for the two focal lengths, respectively, both approaching their theoretical diffraction limits (830 nm and 1298 nm, calculated as 0.61 λ/NA, where λ = 589 nm and NA = 0.43 and 0.28). These results confirm that the metalens achieves near-diffraction-limited focusing, demonstrating its high-resolution imaging capability.

Furthermore, the final focusing efficiency of the metalens at 30 µm, defined as the product of the FDTD-derived transmission (33.1%) and the scalar diffraction focusing efficiency (70%), is therefore 23.2% and the final overall efficiency is 33.2% × 86.1% = 28.5% at 50 µm. The high focusing efficiencies derived from the scalar diffraction analysis (70% and 86.1%) confirm the exceptional accuracy of our metalens design in engineering the desired wavefront.

**Figure 5:**
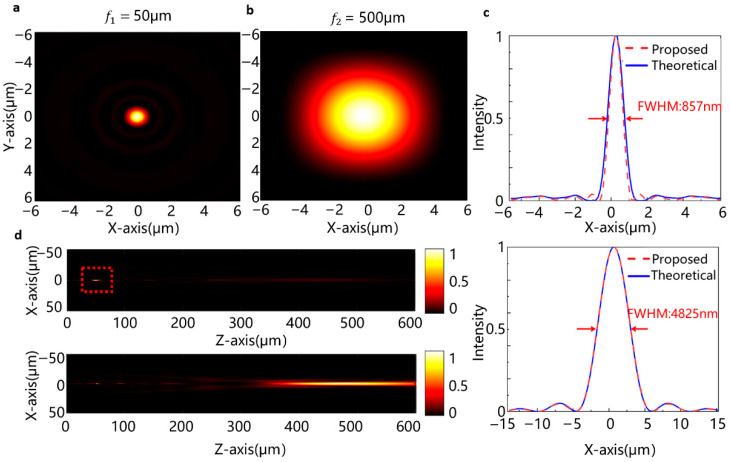
Simulation results at focal length 50 μm and 500 μm. (**a**) Intensity profile of the focused spot at focal 50 μm; (**b**) intensity profile of the focused spot at focal 500 μm in the two-dimensional plane (X-Y axis); (**c**) cross-sectional profiles of the focused spot at focal 50 μm (top), FWHM = 857 nm, and (bottom) at focal length 500 μm, FWHM = 4825 nm; (**d**) simulated intensity along the z-axis at focal length 50 μm and 500 μm for (top) H_PEDOT_ = 210 nm and (bottom) the H_PEDOT_ = 330 nm.

To further verify the scalability of our design for multi-focal-length operation, we extended the validation to a metalens with focal lengths of 50 μm and 500 μm, achieving a zoom ratio of 10:1. The metasurface is discretized with a step size of 150 steps, and the metalens radius is set to 27 μm. The large number of nano-unit configurations requires extensive FDTD simulations. Considering the computational resources constraints, we employ the Kirchhoff diffraction formula for the simulations. Simulations are conducted under different applied voltages, and the results are presented in Figure 5a–d. The corresponding XY focal plane intensity distributions clearly reveal well-defined focusing spots at both distances. Among them, Figure 5a corresponds to the light intensity distribution at *f*_1_ under Voltage 1 (PEDOT:PSS thickness is 210 nm), while Figure 5b corresponds to that at *f*_2_ under Voltage 2 (PEDOT:PSS thickness is 330 nm). For incident light with a wavelength of 589 nm, the FWHM is 857 nm and 4825 nm, while the imaging resolution is 756.2 nm and 3993.3 nm, as presented in Figure 5c. Figure 5d displays the light intensity distributions along the z-axis, where the distinct intensity contrast at 50 μm and 500 μm confirms the reliable performance of our algorithmic design across a broad focal length range. The flexibility in accommodating different target focal lengths underscores the adaptability of our optimization framework for diverse application requirements.

## 3. Discussion

In conclusion, we have successfully designed and validated an electrically tunable metalens based on PEDOT:PSS. By leveraging the voltage-dependent swelling property of PEDOT:PSS, the FP cavity length is modulated to produce distinct electromagnetic responses, enabling dynamic zooming functionality. A comprehensive library of Au nano-units has been constructed to achieve diverse phase profiles. The nano-units arrangement is optimized using the PSO algorithm, which effectively suppressed inter-unit phase error and ensured high phase modulation accuracy. Simulation results confirm that the metalens achieves near-diffraction-limited focusing, with FWHM values of 778 nm and 1027 nm at focal lengths of 30 μm and 50 μm. Furthermore, the design is extended to support tunable focusing with a zoom ratio of up to 10:1. Compared to conventional zoom systems, our tunable metalens reduces the reliance on complex optical components while maintaining high phase control accuracy. We anticipate that this work will open up new avenues for highly integrated, low-cost, and multifunctional dynamic optical systems, and promote their applications in fields such as advanced microscopy, high-resolution imaging, and miniaturized optical displays.

## Data Availability

The data presented in this study are available on request from the corresponding author (The data are not publicly available due to privacy).
